# The Effect of Probiotics, Prebiotics, and Synbiotics on CD4 Counts in HIV-Infected Patients: A Systematic Review and Meta-Analysis

**DOI:** 10.1155/2020/7947342

**Published:** 2020-11-26

**Authors:** Yuan-Sheng Fu, Qin-Shu Chu, Akililu Alemu Ashuro, Dong-Sheng Di, Qi Zhang, Xue-Mei Liu, Yin-Guang Fan

**Affiliations:** ^1^Department of Epidemiology and Biostatistics, School of Public Health, Anhui Medical University, 81 Meishan Road, Hefei, Anhui 230032, China; ^2^Liuzhou Center for Disease Control and Prevention, 1 Tanzhongxi Road, Liuzhou, Guangxi Zhuang Autonomous Region 545000, China

## Abstract

**Background:**

Probiotics as a potential adjuvant therapy may improve the restoration of the intestinal CD4^+^ T-cell population in HIV-infected patients, whereas findings from clinical trials are inconsistent. This systematic review and meta-analysis of randomized controlled trials (RCTs) was performed to quantify the effects of probiotic, prebiotic, and synbiotic supplementation on CD4 counts in HIV-infected patients.

**Methods:**

We searched PubMed, Embase, Web of Science, Scopus, and the Cochrane Central Register of Controlled Trials for relevant articles published up to March 20, 2020. Two authors independently performed the study selection, data extraction, and risk of bias assessment. Data were pooled by using the random effects model, and weighted mean difference (WMD) was considered the summary effect size. Publication bias was evaluated by a funnel plot and Egger's test.

**Results:**

The search strategy identified 1712 citations. After screening, a total of 16 RCTs with 19 trials were included in the meta-analysis. Pooling of the extracted data indicated no significant difference between the probiotics/prebiotics/synbiotics and placebo groups on CD4 counts (WMD = 3.86, 95% confidence interval (CI) -24.72 to 32.45, *P* = 0.791). In subgroup analysis, a significant increase in CD4 counts was found in the study with high risk of bias (WMD = 188, 95% CI 108.74 to 227.26, *P* ≤ 0.001). Egger's test showed no evidence of significant publication bias (*P* = 0.936).

**Conclusions:**

In summary, the evidence for the efficacy of probiotics, prebiotics, and synbiotics in improving HIV-infected patients' CD4 counts as presented in currently published RCTs is insufficient. Therefore, further comprehensive studies are needed to reveal the exact effect of probiotics, prebiotics, and synbiotics on CD4^+^ cell counts.

## 1. Introduction

Individuals living with human immunodeficiency virus (HIV) are characterized by progressive CD4^+^ T-cell depletion and immunodeficiency [1]. HIV infection alters gut microbial ecology [2], and a huge gastrointestinal (GI) pathology is observed even during primary infection. HIV enteropathy includes pronounced gut-associated CD4^+^ T-cell loss and an impaired gastrointestinal (GI) epithelial barrier [3–5]. These detrimental changes presumably result in microbial translocation and a loss of gut homeostasis [1, 6, 7], which in turn leads to chronic immune activation and disease progression [8, 9]. In addition, the efficacy of antiretroviral treatment in the GI tract seems to be poor, resulting in insufficient reconstitution of CD4^+^ T cells and incomplete viral suppression [10–12]. In view of the key role of decreasing bacterial translocation and proinflammatory cytokine production in the maintenance of gut homeostasis, new therapies aimed at restoring the integrity of the epithelial and gut-associated lymphoid tissue (GALT) through oral prebiotics, probiotics, or synbiotics, as well as improving chronic immune activation, are promising new strategies to alleviate disease progression of HIV patients.

Probiotics are defined as “live microorganisms which, when administered in adequate amounts, confer a health benefit on the host” [13] and have an effect on the immunological response. They mainly stimulate the secretion of polymeric IgA, avoid the overgrowth and translocation of bacteria, and promote the development of regulatory T (Treg) cells through the production of anti-inflammatory cytokines [14–17]. Related to probiotics are prebiotics, indigestible food ingredients, generally oligosaccharides, that improve host health by selectively stimulating the growth of beneficial bacteria in the colon, such as *Bifidobacteria* and *Lactobacilli* [18, 19]. Prebiotics can increase the production of short-chain fatty acids (SCFAs), thereby reducing inflammation [20]. A study in mice also showed that prebiotics had an immunostimulatory effect on the induced site [21]. Synbiotics are products that combine prebiotics and probiotics, with a potentially synergistic action. Given the evidence of the beneficial effects of probiotic, prebiotic, and synbiotic consumption during the course of different viral infections and noninfectious diseases [22–25], a growing body of studies try to prove that the use of probiotics, prebiotics, and synbiotics may be able to help preserve the immune function of HIV patients and consequently prevent the depletion of CD4^+^ T cells. However, the results are inconsistent across different studies [26–29]. Therefore, we conducted a systematic review and meta-analysis of available RCTs to evaluate the effect of probiotics, prebiotics, and synbiotics on CD4 counts in HIV patients.

## 2. Materials and Methods

### 2.1. Search Strategy

This systematic review and meta-analysis was conducted in accordance with the guidelines of the Cochrane Handbook [30] and was reported in compliance with the Preferred Reporting Items for Systematic Reviews and Meta-Analyses (PRISMA) statement [31]. We searched PubMed, Embase, Web of Science, Scopus, and the Cochrane Central Register of Controlled Trials for studies published before March 20, 2020. Studies were searched using the following search terms: (Probiotic OR Prebiotic OR Synbiotic OR *Lactobacillus* OR *Bifidobacterium* OR *Saccharomyces* OR “*Streptococcus thermophiles”* OR “fermented milk” OR “*Escherichia coli”*) AND (HIV/AIDS OR HIV OR AIDS OR “Human Immunodeficiency Virus” OR “Acquired Immunodeficiency Syndrome”) AND (Random OR Randomized OR “Randomized controlled trial” OR “controlled clinical trial” OR “randomized studies”). No restrictions were placed on the language and date. In addition, the references of the included articles were also screened to find other relevant publications.

### 2.2. Study Selection

Studies were included with the following criteria: (1) RCTs with parallel or cross-over design, (2) studies conducted in HIV-1-infected adults over 18 years of age, (3) intervention using probiotics, prebiotics, or synbiotics, (4) comparison with placebo or control groups, and (5) CD4 counts as a primary or secondary outcome. Exclusion criteria were as follows: (1) nonrandomized clinical trials; (2) uncontrolled studies; (3) studies conducted in children or pregnant women; (4) letters, conference abstracts, case reports, reviews, or observational studies; or (5) studies not clearly reporting CD4 counts before or after the intervention. All studies were independently assessed by two authors, and any disagreement was resolved by a third researcher.

### 2.3. Data Extraction and Quality Assessment

The following data were extracted: first author's name, year of publication, study design, country of study, sample size, age and gender of participants, details of interventions (including strain, dosage, and duration of intervention), intake of antiretroviral drugs or not, and the main results on the interested outcomes. For the missing data, the authors were contacted through e-mails to get relevant data. The methodological quality of included studies was evaluated by using the Cochrane Collaboration's risk of bias tool [32]. The following domains were assessed: random sequence generation, allocation concealment, blinding of participants and personnel, blinding of outcome assessment, incomplete outcome data, selective reporting, and other bias. The risk of bias for each domain was judged as low, high, or unclear according to the Cochrane Handbook for Systematic Reviews. Any disagreements during the processes of data extraction and quality assessment were resolved by discussion. When consensus was not reached, a third investigator worked as an arbitrator.

### 2.4. Data Synthesis and Analysis

The mean difference (MD) and standard deviation (SD) of CD4 counts between the probiotics/prebiotics/synbiotics and control groups were used to estimate the pooled effects. For the trials that provided more than one interval results, the last intervention results were included in the analysis. And weighted mean difference (WMD) with 95% confidence interval (CI) was considered the summary effect size. Heterogeneity was assessed by Cochran's *Q* test and *I*-square (*I*^2^) statistic, and heterogeneity with an *I*^2^ value > 50% or *P* < 0.1 was considered significant [33]. To account for heterogeneity between articles, a random effects model was applied in this meta-analysis. The subgroup analysis was also carried out according to the type of intervention, intake of antiretroviral drugs or not, duration of intervention, income of the country, and risk of bias assessment. Furthermore, a funnel plot and Egger's linear regression were used to evaluate the potential publication bias. Meta-analysis was performed using Stata software version 14.0 (Stata Corp., College Station, TX, USA) and RevMan version 5.3 (Cochrane Collaboration, Oxford, UK). A two-tailed *P* < 0.05 was considered to be significant.

## 3. Results

**Figure 1:**
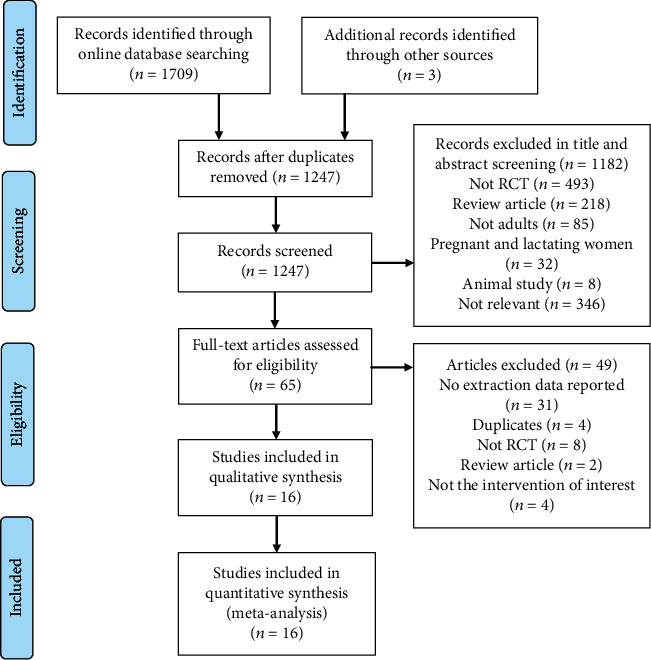
Flowchart of study selection.

A total of 1712 relevant articles were identified by searching the initial online databases. After duplicates were removed, the remaining 1247 studies were screened by title and abstract, 1182 of which were excluded, as they did not meet the eligibility criteria. The full text of the remaining 65 records was retrieved, and 16 studies (19 trials) that fulfilled the inclusion criteria were included in the systematic review and meta-analysis [2, 26–29, 34–44]. The process of study selection and reasons for exclusion are presented in Figure 1.

### 3.1. Characteristics of the Included Studies

The majority of the studies were randomized, double-blind, placebo-controlled trials except one [40] randomized, nonblinded, placebo-controlled trial; one [42] randomized, triple-blind, placebo-controlled trial; and one [39] randomized, double-blind, cross-over placebo-controlled trial. These studies were published from 1998 to 2020, with sample sizes ranging from 10 to 340 individuals. The duration of intervention varied from 15 days to 52 weeks. Twelve trials administered probiotics [2, 26, 28, 34–36, 38–40, 42–44], while four trials administered prebiotics [27, 29, 44] and three trials administered synbiotics [37, 41, 44]. All of the included clinical trials were with two-arm parallel design except two studies [29, 44] which were with three-arm and four-arm parallel design. The three-arm and four-arm parallel design studies were considered two and three trials. The characteristics of the enrolled studies are summarized in Table 1.

### 3.2. Risk of Bias Assessment

**Figure 2:**
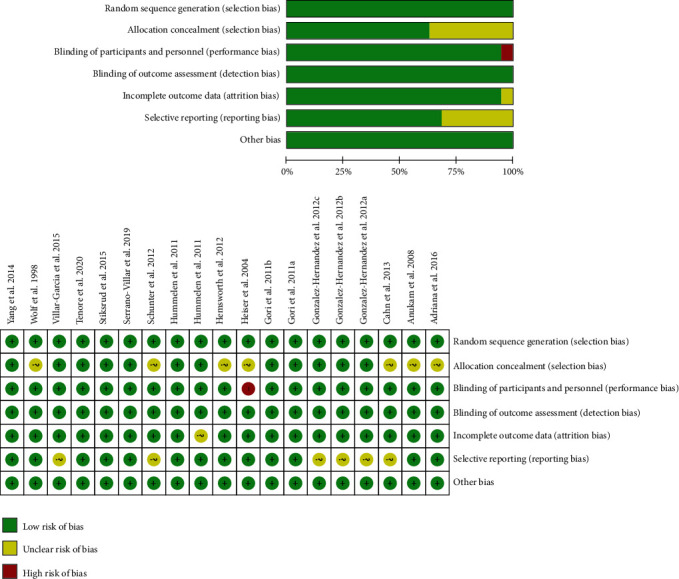
Risk of bias and its summary for the included trials.

The risk of bias of the included studies is presented in Figure 2. Among the 19 trials, seven [26, 28, 29, 34, 37, 38] were judged to have a low risk of bias; eleven [2, 27, 35, 36, 39, 41-44] were categorized as having unclear risk and one [40] as having high risk of bias. All the included trials achieved adequate random sequence generation and blinding of outcome assessment. Seven [2, 27, 39–43] studies provided no description of allocation concealment procedure, and six [27, 36, 41, 44] studies were rated to have unclear risk of selective reporting bias. Attrition bias was found in one study [35] due to loss of participants during the study period. All of the trials except for one [40] had a high risk of bias in blindness of participants and key study personnel.

### 3.3. Meta-Analysis: Main Results

**Figure 3:**
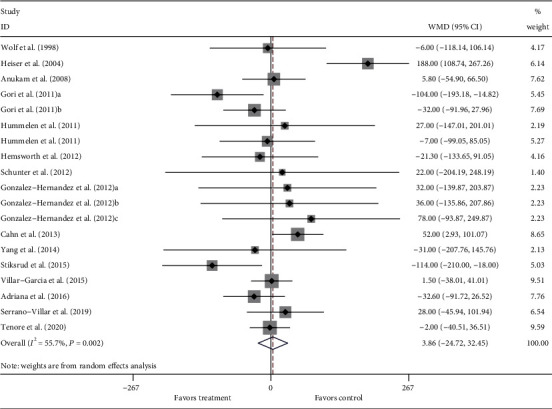
Forest plot of the effect of probiotic, prebiotic, and synbiotic supplementation on CD4 counts. The square in the figure represents the effect of the study, and the size of the square represents the weight of the study. The horizontal line represents the confidence interval of the effect value. The diamond in the figure represents the pooled effect. WMD: weighted mean difference; CI: confidence interval.

In total, 16 RCTs with 19 treatment arms were included in the meta-analysis. Due to the relatively high heterogeneity among the included studies (*I*^2^ = 55.7%, *P* = 0.002), a random effects model was selected for quantitative synthesis. Overall, the pooled results indicated no significant difference after probiotic, prebiotic, and synbiotic supplementation in comparison with the placebo controls on CD4 counts (WMD = 3.86, 95% CI: –24.72 to 32.45, *P* = 0.791). The forest plot of the meta-analysis is shown in Figure 3.

### 3.4. Subgroup Analysis

Because of the existence of heterogeneity, subgroup analysis was conducted based on the type of intervention (probiotics *vs*. prebiotics *vs*. synbiotics), duration of intervention (<30 *vs*. ≥30 days), intake of antiretroviral drugs or not (yes *vs.* no), income of country (high *vs*. low and middle), and risk of bias assessment (low *vs*. unclear *vs*. high). The result of subgroup analysis for trials with high risk of bias showed a significant increase in the CD4 counts compared to that with low and unclear risk of bias (WMD = 188; 95% CI, 108.74, 227.26; *P* ≤ 0.001). However, the other subgroup analysis revealed that none of the subgroups achieved statistical significance. A summary of the results of subgroup analysis is shown in Table 2.

### 3.5. Sensitivity Analysis and Publication Bias

**Figure 4:**
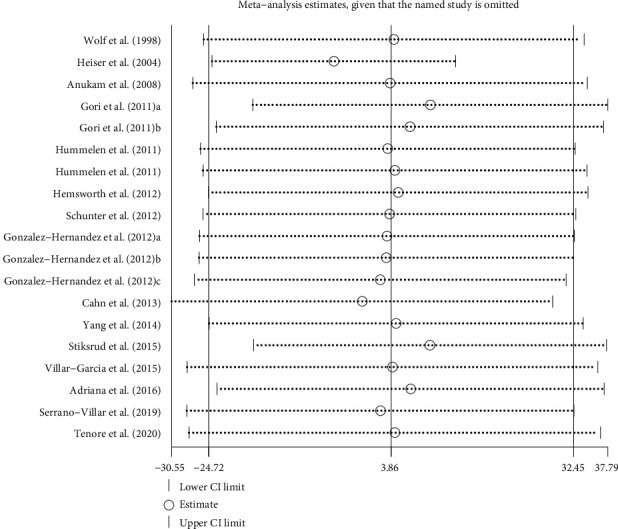
Sensitivity analysis of probiotic, prebiotic, and synbiotic supplementation on CD4 counts.

**Figure 5:**
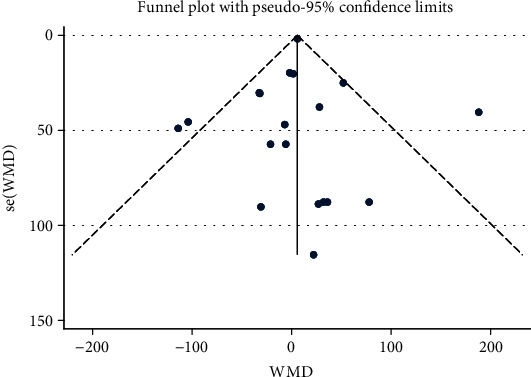
Funnel plot to test the publication bias in the included studies.

Sensitivity analysis was carried out by removing studies one by one to test the reliability of the results of meta-analysis. The results in Figure 4 showed that no matter which study was omitted, the overall statistical significance does not change. In addition, excluding these studies that provide more than one interval results does not change the significance of the findings (WMD = 4.28, 95% CI: –30.88 to 39.44, *P* = 0.81). Publication bias was assessed by a funnel plot and the result of Egger's test. Visual inspection of the funnel plot showed that RCTs are symmetrically scattered around the null vertical line, suggesting no bias (Figure 5). Egger's regression intercept test confirmed that there was no significant publication bias (*P* = 0.936).

## 4. Discussion

In this study, we reviewed and performed a systematic review and meta-analysis to assess the effect of probiotic, prebiotic, and synbiotic supplementation on CD4 counts in HIV-infected patients. The results of our meta-analysis show that these interventions did not cause any significant change on the CD4 counts. In subgroup analysis, a significant increase in CD4 counts was found in studies with high risk of bias. However, subgroup analysis based on the type of intervention, intake of antiretroviral drugs or not, duration of intervention, and the income of the country of the included studies revealed no significant findings. Egger's test showed that the potential risk of publication bias is low, and sensitivity analysis supports the reliability of the results.

These findings are counterintuitive because they appear to be inconsistent with some previous studies [17, 27, 45]. HIV infection dramatically alters the intestinal environment, leading to significant changes in the structural and functional characteristics of the intestinal tract, including microbial translocation and gut inflammation [46–49]. Probiotics, by inhibiting pathogenic bacteria and toxin production, promote gut homeostasis [50]. Therefore, it is expected that probiotic, prebiotic, or synbiotic administration may increase CD4 counts by modulating the gut microbial ecology of HIV patients. However, our study found no significant difference between probiotics/prebiotics/synbiotics and placebo groups in improving CD4 counts. One possible explanation is that in our meta-analysis, the absolute CD4^+^ T cell was reported as a predictor of immune status and disease progression and to be used in quantitative synthesis; however, the CD4^+^ percentage of total T cells as a strong independent predictor of immune status and disease progression [51] may be a more appropriate indicator for comparison. In addition, probiotics are not pharmaceutical substances. Probiotics can be administered as single strains or combination compounds, but different strains produce varied effects and how the single strains interact when coadministered was unclear. Moreover, the dose-response curves of most strains have not been described [52]. In summary, the heterogeneity of probiotic application and the limitations in medicine have hampered the scientific quality of clinical research on probiotics.

Though the interesting outcome in this review is CD4 counts, several included studies [28, 29, 36, 38, 39, 41] also reported related results, namely, gut inflammation and microbial translocation levels, both of them were known to be associated with the progression and prognosis of HIV infection [8–10]. It should be mentioned that many of the trials reported only an improvement in one or two markers of inflammation, while there was no significant difference in the rest of the analysis. Similarly, very few of the studies [2, 26, 28, 37, 41, 43] have evaluated the level of immune markers such as CD8 counts and the CD4/CD8 ratio, which has been considered a prognostic parameter of non-AIDS morbidity [53, 54]. In summary, since the specific mechanism of probiotics in the gut repair is not clear, tracking these outcomes with CD4 counts may yield new and interesting findings, which may provide a broader perspective on the therapeutic potential of probiotics, prebiotics, and synbiotics in HIV patients.

Subgroup analysis revealed that risk of bias assessment may be the source of heterogeneity. However, other subgroup analysis failed to explain the heterogeneity between studies. Also, it should be mentioned that the effects of prebiotic, probiotic, and synbiotic intervention on CD4 counts were statistically significant in trials with high risk of bias. Since only one study was included in the subgroup with high risk of bias and the quality of the study was relatively low due to the failure of blind implementation, the results were hampered with uncertainty.

Our results are different from previously published systematic reviews [55, 56]. Of note, two of the included studies reported improvement of CD4 counts among those receiving probiotic supplements [38, 42]; however, after analysis according to the data inclusion criteria of our meta-analysis, the result showed no significant difference. In addition, compared to previous reviews, our analysis specifically focused on RCTs and adult patients (≥18 years), and we performed a more comprehensive analysis on available evidences that may potentially be involved in the efficacy of probiotic administration on CD4 counts in HIV-infected patients. Our meta-analysis also included the updated references that have not been analyzed in other meta-analysis [26, 27, 37, 43, 57]. These reasons may cause our findings to be inconsistent with other reviews. This meta-analysis has some limitations. First, most of the included trials had relatively small sample sizes, which may lead to an underestimation of the intervention effect; therefore, large-scale trials are warranted. Second, heterogeneity exists between studies in regard to applied probiotic strain(s) and dosage. Therefore, future studies with more high-quality trials are recommended to determine the ideal number and combination of species or strains and their ideal dose for use in probiotic supplements. Third, different formations of administration (yogurt, milk, and capsule) were used in the included trials. Though in vitro analysis of the activity of the probiotics from yogurt or capsules did not differ [34], there may be discrepancies in the survival and colonization of probiotic strains in the intestinal tract.

## 5. Conclusion

In summary, the results of this meta-analysis suggest that the evidence for the efficacy of probiotics, prebiotics, and synbiotics in improving HIV-infected patients' CD4 counts from current RCTs is insufficient. The promotion of these interventions for the benefit of HIV-infected patients in clinical subjects should be implemented only when more valid evidence in this area is obtained. Future clinical studies with a well design and large sample size are needed to further elucidate probiotics, prebiotics, and synbiotics' mechanisms of action, safety profile, and clinical potential, in both support immune system reconstitution and longer-term health outcomes on HIV-infected patients.

## Figures and Tables

**Table 1:** Characteristics of the included randomized controlled trials.

Study (year)	Study design	Country	Sample size	Age (years)	Sex	Duration	ARV	Intervention (strain and daily dose)	Main outcome measures
Wolf et al. (1998)	Randomized, double-blind, placebo-controlled trial	USA	35	23 to 50	M (95%)	21 d	Not on ARV	*Lactobacillus reuteri* (10^10^ cfu/day)	Serum chemistry, hematology, immune profile, urinalysis, physical examination

Heiser et al. (2004)	Randomized, controlled trial	USA	35	42.6 ± 7.4	M (100%)	12 wk	All on ARV	*Acidophilus* and *Bifidobacteria* (1.2 g/d) and soluble fiber (Proctor & Gamble, Cincinnati, 11 g/d)	Diarrhea, CD4 count, HIV RNA

Anukam et al. (2008)	Randomized, triple-blind, placebo-controlled trial	Nigeria	23	18 to 44	F (100%)	15 d	Not on ARV	Probiotic yogurt containing *Lactobacillus rhamnosus* GR-1 and *Lactobacillus reuteri* RC-14 (2.5 × 10^9^ cfu/day)	Hematologic profiles, CD4 count, QoL

Gori et al. (2011a)	Randomized, double-blind, placebo-controlled trial	Italy	31	38.3 ± 9.5	M (66%)	12 wk	ARV naive	Short chain galactooligosaccharides/long-chain fructooligosaccharides/pectin hydrolysate-derived acidic oligosaccharides (15 g/d)	Gut microbiota composition, immunological markers, LPS, sCD14, NK cell activity

Gori et al. (2011b)	Randomized, double-blind, placebo-controlled trial	Italy	33	38.3 ± 9.5	M (76%)	12 wk	ARV naive	Short-chain galactooligosaccharides/long-chain fructooligosaccharides/pectin hydrolysate-derived acidic oligosaccharides (30 g/d)	Gut microbiota composition, immunological markers, LPS, sCD14, NK cell activity

Hummelen et al. (2011)	Randomized, double-blind, placebo-controlled trial	Tanzania	53	NA	F (100%)	25 wk	Not on ARV	*Lactobacillus rhamnosus* GR-1 and *Lactobacillus reuteri* RC-14 (2 × 10^9^ cfu/day)	CD4 count, immune markers (IgG, IgE, IFN-*γ*, and IL-10)

Hummelen et al. (2011)	Randomized, double-blind, placebo-controlled trial	Tanzania	111	NA	F (86%)	4 wk	ARV naive	*Lactobacillus rhamnosus* GR-1 (12.5 × 10^10^ cfu/day) and micronutrients	CD4 count, hematology indicators

Hemsworth et al. (2012)	Randomized, double-blind, cross-over controlled trial	Canada	42	47.6 ± 9.3	M (75%)	30 d	All on ARV	Yogurt containing micronutrients and *Lactobacillus rhamnosus* CAN-1 (minutes 10^9^ cfu/mL)	Immunologic parameters, nutritional and biochemical parameters

Schunter et al. (2012)	Randomized, double-blind, placebo-controlled trial	USA	27	47.5	M (100%)	4 wk	All on ARV	A synbiotic consists of 4 strains of probiotic bacteria (10^10^ each) plus 4 nondigestible, fermentable dietary fibers (2.5 g each)	Bacterial translocation, CD4^+^ T-cells, CD8^+^ T-cells, CRP, sCD14

Gonzalez-Hernandez et al. (2012a)	Randomized, double-blind, placebo-controlled trial	Mexico	10	18 to 65	M (90%)	16 wk	ARV naive	*Lactobacillus rhamnosus* HN001 plus *Bifidobacterium lactis* Bi-07 at 10^9^ cfu/mL	Safety, QoL, CD4 count, cytokine level
Gonzalez-Hernandez et al. (2012b)	Randomized, double-blind, placebo-controlled trial	Mexico	10	18 to 65	M (90%)	16 wk	ARV naive	10 g fructooligosaccharides (FOS)	Safety, QoL, CD4 count, cytokine level

Gonzalez-Hernandez et al. (2012c)	Randomized, double-blind, placebo-controlled trial	Mexico	10	18 to 65	M (100%)	16 wk	ARV naive	(*Lactobacillus rhamnosus* HN001 plus *Bifidobacterium lactis* Bi-07 at 10^9^ cfu/mL)+10 g FOS	Safety, QoL, CD4 count, cytokine level

Cahn et al. (2013)	Randomized, double-blind, controlled trial	Italy, Netherlands, UK, Thailand, US, Brazil, Argentina, Australia	340	39.6	M (82%)	52 wk	Not on ARV	Oligosaccharides (short-chain GOS, long-chain FOS, and pectin-derived AOS) and micronutrients	CD4 count, plasma viral load, safety, and tolerability

Yang et al. (2014)	Randomized, double-blind, placebo-controlled trial	USA	17	49.6 ± 8.7	M (94%)	90 d	All on ARV	*Bacillus coagulans* GBI-30, 6086 (2 × 10^9^ cfu/day)	CD4 count, CD4 percentage, proinflammatory blood biomarkers

Stiksrud et al. (2015)	Randomized, double-blind, placebo-controlled trial	Norway, Sweden	24	50.8	M (67%)	8 wk	All on ARV	Fermented skimmed milk supplemented with *Lactobacillus rhamnosus* GG (10^8^ cfu/mL), *Bifidobacterium animalis* subsp. *Lactis* B-12 (10^8^ cfu/mL), and *Lactobacillus acidophilus* La-5 (10^7^ cfu/mL)	CD4 count,CD4/CD8 ratio, soluble inflammation markers, D-dimer, LPS, sCD14

Villar-Garcia et al. (2015)	Randomized, double-blind, placebo-controlled trial	Spain	44	47.5	M (84%)	12 wk	All on ARV	*Saccharomyces boulardii* (2 capsules 3 times a day or 6 × 10^7^ living bacteria)	Microbial translocation and inflammation markers, immunological and clinical data

Adriana et al. (2016)	Randomized, double-blind, placebo-controlled trial	USA	73	51	M (86%)	22 wk	All on ARV	Probiotic Visbiome Extra Strength	sCD14, IL-6, CD4 count, CD4/CD8 ratio, sCD163

Serrano-Villar et al. (2019)	Randomized, double-blind, placebo-controlled trial	Spain	59	38	M (92%)	48 wk	ARV naive	PMT25341 (a mixture of prebiotics, probiotics, oligonutrients, essential amino acids, omega-3 fatty acids)	CD4 count, CD4/CD8 ratio, markers of T-cell activation, bacterial translocation, inflammation

Tenore et al. (2020)	Randomized, double-blind, placebo-controlled trial	Brazil	48	44.5	M (90%)	12 wk	All on ARV	*Lactobacillus casei* Shirota	CD4 count, CD4/CD8 ratio, levels of CD4^+^ and CD8^+^ T-cell activation, sCD14

NA: not available; ARV: antiretroviral; F: female; M: male; d: day; wk: week; NK: natural killer; LPS: lipopolysaccharide; IFN-*γ*: interferon-*γ*; QoL: quality of life; IL-10: interleukin-10; CRP: C-reactive protein.

**Table 2:** Summary of subgroup analysis.

Subgroup	No. of trials	WMD	95% CI	*P*	Weight	*I* ^2^ (%)	*P* for heterogeneity	*P* for subgroup difference
Intervention type								0.60
Probiotics	12	4.23	(-33.02, 41.47)	0.824	65.80	61.6	0.003	
Prebiotics	4	-13.80	(-87.87, 60.28)	0.715	24.30	72.2	0.013	
Synbiotics	3	34.67	(-30.38, 99.72)	0.296	10.17	0.0	0.866	
Duration								0.91
<30 days	4	1.44	(-43.81, 46.68)	0.950	18.46	0.0	0.992	
≥30 days	15	4.57	(-30.48, 39.62)	0.798	81.54	65.5	≤0.001	
Intake of ARV								0.83
Yes	9	6.94	(-40.38, 54.26)	0.774	52.25	72.3	≤0.001	
No	10	0.81	(-32.18, 33.79)	0.962	47.45	23.7	0.225	
Income of country								0.83
High	4	-1.33	(-44.16, 41.50)	0.952	68.62	72.1	≤0.001	
Low and middle	15	4.37	(-24.52, 33.26)	0.767	31.38	0.0	0.979	
Risk of bias assessment								≤0.001
Low	7	-29.01	(-65.70, 7.69)	0.121	41.70	38.7	0.134	
Unclear	11	9.82	(-12.97, 32.60)	0.399	52.16	0.0	0.800	
High	1	188	(108.74, 227.26)	≤0.001	6.14	NA	NA	

WMD: weighted mean difference; CI: confidence interval; NA: not available; ARV: antiretroviral.

## Data Availability

The data is available upon request.

## Supplementary Materials

Supplementary materials Detailed search strategy. Figure Legends
