# Supplementary material for: The Effect of Probiotics, Prebiotics, and Synbiotics on CD4 Counts in HIV-Infected Patients: A Systematic Review and Meta-Analysis
Source: Biomed Res Int. 2020 Nov 26;2020:7947342. doi: 10.1155/2020/7947342 (PMC7718054; doi:10.1155/2020/7947342)
Supplement: Supplementary materials — Detailed search strategy. Figure Legends [file 7947342.f1.docx]

**Search strategy:**

**PubMed**

**#1** (Probiotics[mh] OR Prebiotics[Mh] OR Synbiotics[mh] OR Lactobacillus[mh] OR Bifidobacterium[mh] OR Saccharomyces[mh] OR Streptococcus thermophilus[mh] OR Escherichia coli[mh] OR Cultured Milk Products[mh] OR Probiotic*[Tiab] OR Prebiotic*[Tiab] OR Synbiotic*[Tiab] OR Lactobacillus[Tiab] OR Bifidobacterium[Tiab] OR Saccharomyces[Tiab] OR Streptococcus thermophilus[Tiab] OR Escherichia coli[Tiab] OR fermented milk[Tiab])

**#2** (HIV[mh] OR Acquired Immunodeficiency Syndrome[mh] OR HIV[Tiab] OR AIDS[Tiab] OR HIV/AIDS[Tiab] OR Human Immunodeficiency Virus[Tiab] OR Acquired Immunodeficiency Syndrome[Tiab])

**#3** ((Randomized controlled trial[pt] OR controlled clinical trial[pt] OR randomized[tiab] OR randomised[tiab] OR placebo[tiab] OR "drug therapy"[sh] OR randomly[tiab] OR trial[tiab] OR groups[tiab])

**#4** **#1 AND #2 AND #3**

**Cochrane Central Register of Controlled Trials (CENTRAL)**

**#1** ([mh Probiotics] OR [mh Prebiotics] OR [mh Synbiotics] OR [mh Lactobacillus] OR [mh Bifidobacterium] OR [mh Saccharomyces] OR [mh "Streptococcus thermophilus"] OR [mh "Escherichia coli"] OR [mh "Cultured Milk Products"] OR Probiotic*:ti,ab OR Prebiotic*:ti,ab OR Synbiotic*:ti,ab OR Lactobacillus:ti,ab OR Bifidobacterium:ti,ab OR Saccharomyces:ti,ab OR "Streptococcus thermophilus":ti,ab OR "Escherichia coli":ti,ab OR "fermented milk":ti,ab)

**#2** ([mh HIV] OR [mh "Acquired Immunodeficiency Syndrome"] OR HIV:ti,ab OR AIDS:ti,ab OR HIV/AIDS:ti,ab OR "Human Immunodeficiency Virus":ti,ab OR "Acquired Immunodeficiency Syndrome":ti,ab)

**#3** **#1 AND #2**

**Embase**

**#1** ('probiotic agent'/exp OR 'prebiotic agent'/exp OR 'synbiotic agent'/exp OR 'Lactobacillus'/exp OR 'Bifidobacterium'/exp OR 'Saccharomyces'/exp OR 'Streptococcus thermophilus'/exp OR 'Escherichia coli '/exp OR 'fermented milk product'/exp OR Probiotic*:ti,ab OR Prebiotic*:ti,ab OR Synbiotic*:ti,ab OR Lactobacillus:ti,ab OR Bifidobacterium:ti,ab OR Saccharomyces:ti,ab OR "Streptococcus thermophilus":ti,ab OR "Escherichia coli":ti,ab OR "fermented milk":ti,ab)

**#2** ('human immunodeficiency virus'/exp OR 'acquired immune deficiency syndrome'/exp OR hiv:ab,ti OR aids:ab,ti OR 'hiv/aids':ab,ti OR 'acquired immunodeficiency syndrome':ab,ti OR 'human immunodeficiency virus':ab,ti)

**#3** (random* OR factorial* OR crossover* OR placebo* OR blind* OR assign* OR allocat* OR 'crossover procedure'/exp OR 'double blind procedure'/exp OR 'randomized controlled trial'/exp OR 'single blind procedure'/exp)

**#4** **#1 AND #2 AND #3**

**Web of Science**

**#1** (Probiotics OR Prebiotics OR Synbiotics OR Lactobacillus OR Bifidobacterium OR Saccharomyces OR “Streptococcus thermophiles” OR “Escherichia coli” OR Probiotic* OR Prebiotic* OR Synbiotic* OR Lactobacillus* OR Bifidobacterium* OR Saccharomyces* OR Streptococcus* OR yoghurt OR “fermented milk”)

**#2** (HIV OR “Acquired Immunodeficiency Syndrome” OR AIDS OR HIV/AIDS OR “Human Immunodeficiency Virus”)

**#3** (TS=(random* OR placebo* OR allocat* OR crossover* OR "cross over" OR ((singl* OR doubl*)NEAR/1 blind*)) OR TI=(trial))

**#4** **#1 AND #2 AND #3**

**Scopus**

**#1** Probiotics OR Prebiotics OR Synbiotics OR Lactobacillus OR Bifidobacterium OR Saccharomyces OR “Streptococcus thermophiles” OR “Escherichia coli” OR Probiotic* OR Prebiotic* OR Synbiotic* OR Lactobacillus* OR Bifidobacterium* OR Saccharomyces* OR Streptococcus* OR yoghurt OR “fermented milk”

**#2** HIV OR “Acquired Immunodeficiency Syndrome” OR AIDS OR HIV/AIDS OR “Human Immunodeficiency Virus”

**#3** random* OR placebo* OR allocat* OR crossover* OR assign* OR blind*OR trial

**#4** **#1 AND #2 AND #3**

**Figure legends:**

**Figure 1.** Preferred Reporting Items for Systematic Reviews and Meta-Analyses flowchart of articles for inclusion in a systematic review of the effect of probiotics, prebiotics, and synbiotics on CD4 counts.

**Figure 2.** Risk of bias and risk of bias summary for included trials. Low at risk of bias: green color and positive symbol; Unclear at risk of bias: yellow color and question symbol; High at risk of bias: red color and negative symbol.

**Figure 3.** Forest plot of the effect of probiotics, prebiotics, and synbiotics supplementation on CD4 counts. A random-effect model was used to analysis the effectiveness of intervention. The square in the figure represents the effect of the study, and the size of the square represents the weight of the study. The horizontal line represents the confidence interval of the effect value. The diamond in the figure represents the pooled effect. WMD, weighted mean difference; CI, confidence interval.

**Figure 4.** Sensitivity analysis of probiotics, prebiotics and synbiotics supplementation on CD4 counts. The middle vertical line represents the pooled effect estimate, the left and right vertical lines represent the upper and lower limits of the pooled effect estimate 95% confidence interval; the horizontal line corresponding to each study indicates the pooled effect and 95% confidence interval of the remaining studies after removing the study. CI, confidence interval.

**Figure 5.** Funnel plot to test the publication bias in the included studies. Visual inspection of the funnel plot showed that RCTs are symmetrically scattered around the null vertical line, suggesting no bias. WMD, weighted mean difference.

**Table 1.** Characteristics of the included randomized controlled trials evaluating the effect of probiotics, prebiotics, and synbiotics on CD4 counts.

**Table 2.** Summary of subgroup analysis of included randomized controlled trials in meta-analysis of the effect of probiotics, prebiotics, and synbiotics on CD4 counts.
